# Molecular Spectrum, Ethnic and Geographical Distribution of Thalassemia in the Southern Area of Hainan, China

**DOI:** 10.3389/fped.2022.894444

**Published:** 2022-06-10

**Authors:** Ying Yu, Chunjiao Lu, Ying Gao, Cuiyun Li, Dongxue Li, Jie Wang, Hui Wei, Zhaohui Lu, Guoling You

**Affiliations:** ^1^Antenatal Diagnostic Center, Sanya Women and Children's Hospital Managed by Shanghai Children's Medical Center, Sanya, China; ^2^Department of Public Health, Sanya Women and Children's Hospital Managed by Shanghai Children's Medical Center, Sanya, China; ^3^Molecular Genetic Diagnosis Center, Sanya Women and Children's Hospital Managed by Shanghai Children's Medical Center, Sanya, China; ^4^Department of Pediatric Surgery, Sanya Women and Children's Hospital Managed by Shanghai Children's Medical Center, Sanya, China; ^5^Department of Cardiothoracic Surgery, Shanghai Children's Medical Center, School of Medicine, Shanghai Jiao Tong University, Shanghai, China; ^6^Department of Laboratory Medicine, Shanghai Children's Medical Center, School of Medicine, Shanghai Jiao Tong University, Shanghai, China

**Keywords:** thalassemia, genetic diagnosis, molecular spectrum, Hainan Province, China

## Abstract

**Background:**

Thalassemia is one of the most common genetic diseases in southern China. Accurate population frequency data regarding the occurrence and distribution of thalassemia are important for designing appropriate prevention strategies for thalassemia. This study aims to reveal the molecular spectrum, ethnic and geographical distribution of thalassemia in the southern area of Hainan Province, China.

**Methods:**

A total of 9813 suspected carriers of thalassemia were screened for genetic analysis by using the PCR-reverse dot blot hybridization method targeting three known deletions of α-thalassemias (--^SEA^, -α^3.7^, and -α^4.2^), three nondeletional mutations of α-thalassaemias (α^CS^, α^QS^, and α^WS^) and the 17 most common mutations of β-thalassaemias in the Chinese population.

**Results:**

Approximately 6,924 subjects were genetically diagnosed as thalassemia carriers or patients, including 5812 cases of α-thalassemia (83.9%), 369 cases of β-thalassemia (5.3%), and 743 cases of α-composite β-thalassemia (10.7%). A total of 21 distinct genotypes were identified among the 5,812 α-thalassemia carriers, -α^4.2^/αα, -α^3.7^/αα, and -α^3.7^/-α^4.2^ were the most common α-thalassemia genotypes. The most frequent β-thalassemia genotype was β^CD41−42^/β^N^, with a notable proportion of 69.6%, followed by the β^−28*M*^/β^N^, β^IVS−II−654^/β^N^, β^CD71−72^/β^N^, β^E^/β^N^, and β^CD17^/β^N^ genotypes. In addition, 37 genotypes were detected among the 743 cases of both α- and β-thalassemia mutations. The α-thalassemia genotypes were most commonly found in the Li people, who accounted for 73.5% of α-thalassemia carriers. The β-thalassemia genotypes were most commonly identified in the Han people, who accounted for 59.4% of β-thalassemia carriers. Among the subjects carrying both α- and β-thalassemia variations, only three ethnic minorities were identified, including the Li, Han, and Miao people, accounting for 82.0, 17.4, and 0.7%, respectively.

**Conclusions:**

Our study indicates that there is high genetic heterogeneity, geographical and ethnic differences in thalassemia in populations in the southern area of Hainan Province. These findings will be helpful in guiding genetic counseling and prenatal diagnosis of thalassemia in Hainan Province.

## Introduction

Thalassemia, also called Mediterranean anemia, was first described by Cooley and Lee in 1925 (1). Thalassemia is a highly clinically heterogeneous group of autosomal recessive hereditary blood disorders, characterized by the reduced or absent synthesis of globin chains, leading to anemia and microcytosis (2). α- and β-Thalassemia are the most common forms of thalassemia, resulting from mutations in the α- and β-globin gene clusters (*HBA1, HBA2*, and *HBB*) on chromosome 16 and chromosome 11, respectively (2). Clinically, thalassemia can be divided into mild (thalassemia gene carriers), intermediate and severe thalassemia according to anemia status (2). Patients with mild thalassemia may show mild hypochromic microcytic anemia, those with intermediate thalassemia show obvious anemia symptoms and may require irregular blood transfusions, and those with severe thalassemia may develop edematous fetuses (α-thalassemia) or developmental jaundice, prognathism, and hepatosplenomegaly. Children with β-thalassemia major tend to die before the age of 5 without treatment. Patients with severe thalassemia depend heavily on blood transfusions to support life, which imposes substantial economic burdens on affected families and society.

It has been recently estimated that there are approximately 350 million carriers of hemoglobinopathies and thalassemias worldwide, of which 270 million are carriers of α-thalassemia and 80–90 million are carriers of β-thalassemia (3). A high prevalence of thalassemia has been mainly reported in tropical and subtropical regions, such as the Mediterranean region, the Middle East, North Africa, India, and Southeast Asia (4). In China, the overall prevalence of α-thalassemia, β-thalassemia, and both α- and β-thalassemia patients was 7.9% (95% confidence interval (CI): 5.54–10.23), 2.2% (95% CI: 1.93–2.48), and 0.5% (95% CI: 0.18–0.79), respectively (5). There were approximately 300,000 patients with severe thalassemia or intermediate thalassemia in China (6). Previous reports indicated a high incidence of thalassemia in southern China, mainly south of the Yangtze River, especially in Guangxi, Guangdong, Hainan, Chongqing, Sichuan, Guizhou, Yunnan, and Fujian Provinces. The incidence of thalassemia is relatively high in Hainan. The carrier rate of thalassemia in the global population is approximately 4.8% (7), while that of thalassemia in Hainan Province is approximately 15%, and could be as high as 55% in the ethnic minority population. As populations in different ethnic and geographical regions have different globin gene mutation spectra, a comprehensive evaluation of the molecular epidemiological characteristics of thalassemia is important for developing appropriate prevention strategies in regions with a high prevalence of thalassemia.

To date, there is no effective treatment for thalassemia, except gene therapy and bone marrow transplantation. To date, several countries have carried out comprehensive national prevention programs, including public education and awareness, carrier screening, genetic counseling, prenatal diagnosis, and preimplantation diagnosis, and have achieved great success in preventing the birth of children with thalassemia major, leading to a consistent decline in the birth rate of children with thalassemia major in some countries such as Greece, Italy, Iran, Pakistan, Singapore, and Thailand (8). In recent years, large-scale surveys of thalassemia have been conducted in different parts of China, including Hainan Province. However, the prevalence of thalassemia is still high. Furthermore, with economic improvement and population migration, thalassemia is spreading to all parts of China, including all parts of Hainan. Only a few studies have reported the prevalence rates and mutations associated with α- and β-thalassemia in this region (9–11), and the current epidemiological characteristics (molecular spectrum, ethnic, and regional) of thalassemia are not completely understood.

In this study, we analyzed α- and β-thalassemia genotypes in patients who underwent thalassemia screening (routine blood tests, hemoglobin electrophoresis) and genetic detection in the southern area of Hainan Province. The results of this study will reveal the detailed molecular spectrum, ethnic and geographical distribution of thalassemia, and provide theoretical bases for genetic counseling and preventive measures to reduce the number of cases of severe thalassemia.

## Materials and Methods

### Subjects

The study subjects were couples and children who underwent thalassemia testing at the Antenatal Diagnostic Centre of Sanya Women and Children's Hospital from September 2019 to September 2021. If the mean corpuscular volume (MCV) was <82 fl and/or the mean corpuscular hemoglobin (MCH) level was <27 pg, subjects were considered suspected carriers of thalassemia. Suspected thalassemia subjects underwent genetic analysis.

### Hematological Analysis

Peripheral blood from each subject was collected in ethylenediaminetetraacetic acid (EDTA) anticoagulant vacutainer blood collection tubes. Hematological data were determined according to standard laboratory procedures using an automated hematology analyzer (Beckman Coulter, Krefeld, Germany).

### Molecular Diagnosis of α- and β-Thalassemia

Genomic DNA was extracted from peripheral blood samples using a Nucleic Acid Extraction Kit (Yaneng Biosciences, Shenzhen, China) following the manufacturer's instructions. A spectrophotometer (NanoDrop, USA) was used to determine the purity and concentration of the gDNA in the sample.

An α/β-thalassemia gene detection kit (PCR-reverse dot blot hybridization method, Yaneng Biosciences, Shenzhen, China) was used to detect three known deletions of α-thalassaemias (--^SEA^, -α^3.7^, and -α^4.2^), three nondeletional mutations of α-thalassaemias [α^ConstantSpring^ (α^CS^), α^QuongSze^ (α^QS^), and α^Westmead^ (α^WS^)] and the 17 most common mutations of β-thalassemia in the Chinese population [CD41/42 (-TCTT), CD43 (G > T), IVS-II-654 (C > T),−28 (A > G),−29 (A > G),−30 (T > C),−32 (C > A), CD71/72 (+A), CD26 (G > A), CD17 (A > T), CD31 (-C), CD14/15 (+G), CD27/28 (+C), IVS-I-1 (G > T), IVS-I-5 (G > C), 5'-UTR;Cap+40-43 (-AAAC), Initiation condon (ATG > AGG)]. The amplified products were analyzed by 1% agarose gel electrophoresis. The assay was performed according to the manufacturer's instructions. The PCR reaction conditions were as follows: initial denaturation at 50°C for 15 min, 94°C for 10 min, denaturation at 94°C for 1 min, annealing at 55°C for 30 s, extension at 72°C for 30 s, 35 cycles of amplification, and extension at 72°C for 5 min. The amplified products were reverse hybridized with the reverse dot-blot membrane of the specific labeled probe. After membrane washing, the signal on the membrane was determined.

## Results

### Genotypes and Mutation Spectrum of Thalassemia

A total of 9,813 suspected carriers of thalassemia underwent genetic analysis. A total of 6,924 subjects were genetically diagnosed as thalassemia positive based on the PCR-reverse dot blot hybridization technique, accounting for 70.56% of all suspected carriers of thalassemia, including 5,812 cases of α-thalassemia (83.9%), 369 cases of β-thalassemia (5.3%), and 743 cases of α-composite β-thalassemia (10.7%). Among the 6,924 patients with thalassemia, 52.8% were females, and the ages of these patients ranged from 1 month old to 60 years old, with a median age of 29 years old.

A total of 71 genotypes were identified in our cohort (Tables 1–3). The mean hematological values for Hb, MCV, and MCH for the different thalassemia genotypes with a frequency of ≥ 5 are displayed in Supplementary Tables 1–3. We identified 21 distinct genotypes among 5,812 α-thalassemia carriers in this study (Table 1). Most of the α-thalassemia carriers were identified as having heterozygous or homozygous deletions. The -α^4.2^/αα deletion was the most common α-thalassemia genotype in the southern area of Hainan, accounting for 18.4% of all α-thalassemia genotypes. The next five most common deletions were -α^3.7^/αα, -α^3.7^/-α^4.2^, and -α^3.7^/-α^3.7^, --^SEA^/αα and -α^4.2^/-α^4.2^ with frequencies of 17.9, 13.0, 6.5, 6.0, and 5.6%, respectively (Table 1). In total, these six deletion genotypes accounted for 67.4% of all α-thalassemia genotypes. The remaining mutations mainly comprised point mutations or point mutations compounded with deletions. α^WS^α/αα was the most common point mutation genotype, present in 10.4% of α-thalassemia carriers. α^WS^α/-α^3.7^ and α^WS^α/-α^4.2^ were identified in 7.3% and 6.1% of α-thalassemia carriers, respectively. Alpha thalassemia intermedia with -α^3.7^/--^SEA^ (52 cases) and -α^4.2^/--^SEA^ (50 cases) was observed in 0.9% and 0.9% of α-thalassemia carriers, respectively.

**Table 1:** Distribution of α-thalassemia genotypes in the southern area of Hainan.

**Genotype**	**Phenotype**	**Total**		**Lingshui**		**Ledong**		**Sanya**		**Baoting**		**Wuzhishan**	
		**Count**	**Ratio (%)**	**Count**	**Ratio (%)**	**Count**	**Ratio (%)**	**Count**	**Ratio (%)**	**Count**	**Ratio (%)**	**Count**	**Ratio (%)**
-α^4.2^/αα	α^+^/α	1,072	18.4	325	20.1	257	16.2	308	21.1	91	14.9	91	16.9
-α^3.7^/αα	α^+^/α	1,039	17.9	287	17.8	255	16.0	275	18.8	124	20.4	98	18.2
-α^3.7^/-α^4.2^	α^+^/α^+^	754	13.0	157	9.7	240	15.1	206	14.1	102	16.7	49	9.1
α^WS^/αα	α^+^/α	607	10.4	178	11.0	158	9.9	137	9.4	55	9.0	79	14.7
α^WS/^-α^3.7^	α^+^/α^+^	422	7.3	122	7.6	129	8.1	78	5.3	43	7.1	50	9.3
-α^3.7^/-α^3.7^	α^+^/α^+^	379	6.5	88	5.5	119	7.5	94	6.4	41	6.7	37	6.9
α^WS^/-α^4.2^	α^+^/α^+^	353	6.1	104	6.4	108	6.8	67	4.6	37	6.1	37	6.9
--^SEA^/αα	α^0^/α	351	6.0	124	7.7	84	5.3	80	5.5	39	6.4	24	4.5
-α^4.2^/-α^4.2^	α^+^/α^+^	324	5.6	74	4.6	108	6.8	90	6.2	29	4.8	23	4.3
α^QS/^αα	α^+^/α	140	2.4	41	2.5	31	2.0	42	2.9	14	2.3	12	2.2
α^WS^/α^WS^	α^+^/α^+^	116	2.0	33	2.0	37	2.3	24	1.6	8	1.3	14	2.6
-α^3.7^/--^SEA^	α^+^/α^0^	52	0.9	24	1.5	13	0.8	10	0.7	4	0.7	1	0.2
-α^4.2^/--^SEA^	α^+^/α^0^	50	0.9	12	0.7	19	1.2	10	0.7	3	0.5	6	1.1
α^QS^/-α^3.7^	α^+^/α^+^	49	0.8	10	0.6	12	0.8	16	1.1	6	1.0	5	0.9
α^QS^/-α^4.2^	α^+^/α^+^	44	0.8	13	0.8	10	0.6	13	0.9	4	0.7	4	0.7
α^QS^/α^WS^	α^+^/α^+^	21	0.4	7	0.4	4	0.3	2	0.1	5	0.8	3	0.6
α^WS^/--^SEA^	α^+^/α^0^	20	0.3	10	0.6	3	0.2	3	0.2	1	0.2	3	0.6
α^CS^/αα	α^+^/α	13	0.2	4	0.2	1	0.1	2	0.1	3	0.5	3	0.6
α^CS^/--^SEA^	α^+^/α^0^	3	0.1	0	0	0	0	3	0.2	0	0	0	0
α^CS/^-α^4.2^	α^+^/α^+^	2	0.03	1	0.1	0	0	1	0.1	0	0	0	0
α^QS^/--^SEA^	α^+^/α^0^	1	0.02	0	0	1	0.1	0	0	0	0	0	0
Total	-	5,812	100	1,614	27.8	1,589	27.3	1,461	25.1	609	10.5	539	9.3

**Table 2:** Distribution of β-thalassemia genotypes in the southern area of Hainan.

**Genotype**	**Phenotype**	**Total**		**Lingshui**		**Ledong**		**Sanya**		**Baoting**		**Wuzhishan**	
		**Count**	**Ratio (%)**	**Count**	**Ratio (%)**	**Count**	**Ratio (%)**	**Count**	**Ratio (%)**	**Count**	**Ratio (%)**	**Count**	**Ratio (%)**
β^41−42*M*^/β^N^	β^0^/β^N^	257	69.6	57	64.8	68	70.8	82	73.2	25	64.1	25	73.5
β^−28*M*^/β^N^	β^+^/β^N^	28	7.6	9	10.2	8	8.3	7	6.3	4	10.3	0	0.0
β^654M^/β^N^	β^+^/β^N^	23	6.2	2	2.3	5	5.2	10	8.9	4	10.3	2	5.9
β^71−72*M*^/β^N^	β^0^/β^N^	17	4.6	5	5.7	6	6.3	4	3.6	1	2.6	1	2.9
β^E^/β^N^	β^+^/β^N^	17	4.6	11	12.5	1	1.0	2	1.8	0	0.0	3	8.8
β^17M^/β^N^	β^0^/β^N^	14	3.8	0	0.0	5	5.2	3	2.7	5	12.8	1	2.9
β^IntM^/β^N^	β^0^/β^N^	3	0.8	1	1.1	1	1.0	0	0	0	0	1	2.9
β^IVS−I−1M^/β^N^	β^0^/β^N^	3	0.8	0	0	0	0	2	1.8	0	0	1	2.9
β^27/28M^/β^N^	β^0^/β^N^	2	0.5	0	0	0	0	2	1.8	0	0	0	0
β^−29*M*^/β^N^	β^+^/β^N^	2	0.5	1	1.1	1	1.0	0	0	0	0	0	0
β^43M^/β^N^	β^0^/β^N^	1	0.3	1	1.1	0	0	0	0	0	0	0	0
β^CAPM^/β^N^	β^+^/β^N^	1	0.3	0	0	1	1.0	0	0	0	0	0	0
β^41−42M^/β^41−42M^	β^0^/β^0^	1	0.3	1	1.1	0	0	0	0	0	0	0	0
Total	–	369	100	88	23.8	96	26.0	112	30.4	39	10.6	34	9.2

**Table 3:** Distribution of α-composite β-thalassemia genotypes in the southern area of Hainan.

**Genotype**	**Total**		**Lingshui**		**Ledong**		**Sanya**		**Baoting**		**Wuzhishan**	
	**Count**	**Ratio (%)**	**Count**	**Ratio (%)**	**Count**	**Ratio (%)**	**Count**	**Ratio (%)**	**Count**	**Ratio (%)**	**Count**	**Ratio (%)**
-α^3.7^/αα & β^41−42M^/β^N^	139	18.7	42	22.6	26	13.4	34	18.6	22	21.2	15	19.7
-α^4.2^/αα & β^41−42*M*^/β^N^	139	18.7	34	18.3	34	17.5	41	22.4	20	19.2	10	13.2
-α^3.7^/-α^4.2^ & β^41−42*M*^/β^N^	99	13.3	22	11.8	28	14.4	26	14.2	12	11.5	11	14.5
α^WS^/αα & β^41−42*M*^/β^N^	92	12.4	25	13.4	28	14.4	18	9.8	11	10.6	10	13.2
α^WS^/-α^3.7^ & β^41−42*M*^/β^N^	51	6.9	10	5.4	14	7.2	7	3.8	8	7.7	12	15.8
α^WS^/-α^4.2^ & β^41−42*M*^/β^N^	51	6.9	11	5.9	16	8.2	11	6.0	6	5.8	7	9.2
-α^4.2^/-α^4.2^ & β^41−42*M*^/β^N^	47	6.3	7	3.8	14	7.2	17	9.3	6	5.8	3	3.9
-α^3.7^/-α^3.7^ & β^41−42*M*^/β^N^	44	5.9	11	5.9	9	4.6	10	5.5	11	10.6	3	3.9
--^SEA^/αα & β^41−42*M*^/βN	12	1.6	2	1.1	8	4.1	0	0	1	1.0	1	1.3
α^QS^/-α^3.7^ & β^41−42*M*^/β^N^	10	1.3	2	1.1	2	1.0	3	1.6	1	1.0	2	2.6
α^WS^/α^WS^ & β^41−42*M*^/β^N^	8	1.1	2	1.1	3	1.5	2	1.1	0	0	1	1.3
α^QS^ /-α^4.2^ & β^41−42*M*^/β^N^	5	0.7	2	1.1	0	0	1	0.5	2	1.9	0	0
α^QS^/α^WS^ & β^41−42*M*^/β^N^	5	0.7	3	1.6	0	0	2	1.1	0	0	0	0
α^QS^ /αα & β^41−42*M*^/βN	4	0.5	1	0.5	1	0.5	1	0.5	1	1.0	0	0
-α^3.7^/--^SEA^ & β^41−42*M*^/β^N^	3	0.4	1	0.5	0	0	0	0	2	1.9	0	0
-α^4.2^/αα & β^71−72*M*^/βN	3	0.4	0	0	1	0.5	2	1.1	0	0	0	0
α^WS^/--^SEA^ & β^41−42*M*^/β^N^	3	0.4	1	0.5	1	0.5	1	0.5	0	0	0	0
α^WS^/αα & β^−28*M*^/β^N^	3	0.4	2	1.1	0	0	0	0	0	0	1	1.3
--^SEA^/αα & β^17M^/β^N^	2	0.3	0	0	1	0.5	0	0	1	1.0	0	0
--^SEA^/αα & β^71−72*M*^/β^N^	2	0.3	0	0	2	1.0	0	0	0	0	0	0
-α^3.7^/αα & β^71−72*M*^/β^N^	2	0.3	0	0	1	0.5	1	0.5	0	0	0	0
-α^4.2^/--^SEA^ & β^41−42*M*^/β^N^	2	0.3	2	1.1	0	0	0	0	0	0	0	0
-α^4.2^/αα & β^654M^/β^N^	2	0.3	1	0.5	1	0.5	0	0	0	0	0	0
α^CS^/αα & β^41−42*M*^/β^N^	2	0.3	0	0	1	0.5	1	0.5	0	0	0	0
-α^3.7^/αα & β^41−42M^/β^41−42M^	2	0.3	3	1.6	0	0	0	0	0	0	0	0
-α^3.7^/αα & β^17M^/β^N^	1	0.1	0	0	1	0.5	0	0	0	0	0	0
-α^3.7^/αα & β^−28*M*^/β^N^	1	0.1	1	0.5	0	0	0	0	0	0	0	0
-α^3.7^/αα & β^43M^/β^N^	1	0.1	0	0	0	0	1	0.5	0	0	0	0
-α^3.7^/αα & β^654M^/β^N^	1	0.1	0	0	1	0.5	0	0	0	0	0	0
-α^4.2^/-α^4.2^ & β^71−72*M*^/β^N^	1	0.1	0	0	1	0.5	0	0	0	0	0	0
-α^4.2^/αα & β^IntM^/β^N^	1	0.1	0	0	0	0	1	0.5	0	0	0	0
α^WS^/-α^3.7^ & β^71−72*M*^/β^N^	1	0.1	0	0	0	0	1	0.5	0	0	0	0
α^WS^/αα & β^654M^/β^N^	1	0.1	1	0.5	0	0	0	0	0	0	0	0
α^WS^/αα & β^71−72*M*^/β^N^	1	0.1	0	0	0	0	1	0.5	0	0	0	0
α^WS^/α^QS^ & β^41−42*M*^/β^N^	1	0.1	0	0	0	0	1	0.5	0	0	0	0
-α^4.2^/-α^4.2^ & β^41−42M^/β^41−42M^	1	0.1	0	0	0	0	0	0	0	0	0	0
Total	743	100	186	25.0	194	26.1	183	24.6	104	14.0	76	10.2

Six α-thalassemia mutations were identified when calculating the frequencies of a specific mutation in all α mutant chromosomes (allele frequency) (Table 4). The -α^3.7^ mutation was the most frequent, accounting for 36.6% of all α mutant chromosomes. The other high-frequency mutations were -α^4.2^, α^WS^α, --^SEA^, α^QS^α, and α^CS^α with allelic frequencies of 35.1, 19.8, 5.3, 3.0, and 0.2%, respectively (Table 4).

**Table 4:** Allele frequency of α-thalassemia in the southern area of Hainan.

**Mutation**	**HGVS name**	**Type**	**Allele**	**Ratio**	**Han**	**Li**	**Miao**	**Hui**	**Zhuang**	**Dai**	**Yao**	**Tujia**	**Buyi**	**Dong**	**Hani**	**Chuanqingren**
			**(*n*)**	**(%)**	**(%)**	**(%)**	**(%)**	**(%)**	**(%)**	**(%)**	**(%)**	**(%)**	**(%)**	**(%)**	**(%)**	**(%)**
-α^4.2^	AF221717	α^+^	3,323	35.1	18.6	80.8	0.5	0.1	0.03	0	0.03	0	0	0	0	0
-α^3.7^	NG_000006.1: g.34164_37967 del3804	α^+^	3,473	36.6	17.4	81.4	0.7	0.4	0	0.03	0.1	0.03	0	0	0	0
--^SEA^	NG_000006.1: g.26264_45564 del19301	α^0^	501	5.3	63.9	30.9	3.6	0.4	0.8	0	0	0	0	0.2	0.2	0
α^WS^α	HBA2:c.369C > G	α^+^	1,880	19.8	16.6	81.6	1.0	0.6	0.1	0	0.1	0	0	0	0	0
α^QS^α	HBA2:c.377 T > C	α^+^	280	3.0	29.6	69.3	0.7	0	0	0	0	0	0	0	0	0.4
A^CS^α	HBA2:c.427 T > C	α^+^	20	0.2	55.0	25.0	0	0	5.0	0	10	0	5.0	0	0	0
Total	-	-	9,477	100	20.6	78.1	0.8	0.3	0.1	0.01	0.1	0.01	0.01	0.01	0.01	0.01

Among the 369 subjects with β-thalassemia, thirteen different genotypes were identified, including 12 heterozygotes and 1 homozygote (Table 2). However, no compound heterozygotes were identified. The most frequent β-thalassemia genotype was β^CD41−42^/β^N^, with a remarkable proportion of 69.6%, followed by β^−28*M*^/β^N^, β^IVS−II−654^/β^N^, β^CD71−72^/β^N^, β^E^/β^N^, and β^CD17^/β^N^; these six genotypes accounted for 96.5% of all β-thalassemia genotypes (Table 2). Regarding allele frequency, the most frequent mutations were β^CD41−42^, β^−28*M*^, β^IVS−II−654^, β^CD71−72^, β^E^, and β^CD17^, with proportions of 88.0, 2.9, 2.4, 2.4, 1.5, and 1.5%, respectively, accounting for 98.8% of all β mutant chromosomes (Table 5).

**Table 5:** Allele frequency of β-thalassemia in the southern area of Hainan.

**Mutation**	**Allele**	**HGVS name**	**Type**	**Allele**	**Frequency**	**Han**	**Li**	**Miao**	**Zhuang**	**Hui**	**Yao**
				**(*n*)**	**(%)**	**(%)**	**(%)**	**(%)**	**(%)**	**(%)**	**(%)**
β^41−42M^	CD41/42 (-TCTT)	*HBB*:c.126_129delCTTT	β^0^	982	88.0	71.3	97.0	85.7	60.0	40.0	0
β^−28M^	−28 (A > G)	HBB:c.-78A > G	β^+^	32	2.9	7.2	0.7	0	0	40.0	0
β^654M^	IVS-II-654 (C > T)	HBB:c.316-197C > T	β^+^	27	2.4	5.7	0.8	0	0	0	50.0
β^71−72M^	CD71/72 (+A)	HBB:c.216_217insA	β^0^	27	2.4	6.3	0.7	0	0	0	0
β^17M^	CD17 (A > T)	HBB:c.52A > T	β^0^	17	1.5	3.4	0.1	9.5	20.0	0	0
β^E^	CD26(G > A)	HBB:c.79G>A	β^+^	17	1.5	2.9	0.7	0	20.0	0	0
β^IntM^	Initiation condon (ATG > AGG)	HBB:c.2T>G	β^0^	4	0.4	1.1	0	0	0	0	0
β^IVS−I−1M^	IVS-I-1 (G > T)	HBB:c.92 + 1G > T	β^0^	3	0.3	0.3	0	4.8	0	0	50.0
β^27/28M^	CD27/28 (+C)	HBB:c.84_85insC	β^0^	2	0.2	0.3	0	0	0	20.0	0
β^−29M^	−29 (A > G)	HBB:c.-79G > A	β^+^	2	0.2	0.4	0	0	0	0	0
β^43M^	CD43 (G > T)	HBB:c.130G > T	β^0^	2	0.2	0.4	0	0	0	0	0
β^CAPM^	5'-UTR;Cap+40-43 (-AAAC)	HBB:c.-11_-8delAAAC	β^+^	1	0.1	0.3	0	0	0	0	0
Total	-	-	-	1,116	100	31.9	64.8	1.9	0.9	0.4	0.2

In our study, 36 genotypes were determined from the 743 cases of both α and β-thalassemia mutations (Table 3). The top six most frequent genotypes were -α^3.7^/αα combined with β^CD41−42^/β^N^ (18.7%), -α^4.2^/αα combined with β^CD41−42^/β^N^ (18.7%), -α^3.7^/-α^4.2^ combined with β^CD41−42^/β^N^ (13.3%), α^WS^α/αα combined with β^CD41−42^/β^N^ (12.4%), α^WS^α/-α^3.7^ combined with β^CD41−42^/β^N^ (6.9%), and α^WS^α/-α^4.2^ combined with β^CD41−42^/β^N^ (6.9%) (Table 3).

### Geographical Distribution of α- and β-Thalassemia Gene Mutations

We analyzed the geographical distributions of α- and β-thalassemia mutations in five regions of the southern area of Hainan Province. The geographical distributions of α-thalassemia mutations are shown in Table 1. -α^4.2^/αα was the most frequent α-thalassemia genotype in Lingshui, Sanya, and Ledong, while -α^3.7^/αα was the most frequent α-thalassemia genotype in Baoting and Wuzhishan. The geographical distributions of β-thalassemia mutations are shown in Table 2. β^CD41−42^/β^N^ was the most common β-thalassemia genotype in Sanya, Ledong, Lingshui, Baoting, and Wuzhishan. -α^3.7^/αα combined with β^CD41−42^/β^N^ was the most frequent α composite β thalassemia genotype in Lingshui, Baoting, and Wuzhishan, and -α^4.2^/αα combined with β^CD41−42^/β^N^ (18.7%) was the most frequent α-composite β-thalassemia genotype in Sanya and Ledong (Table 3).

### Ethnic Distributions of α- and β-Thalassemia Gene Mutations

In addition to geographical factors, ethnic differences also play an important role in genetic diversity. We analyzed the frequency and spectrum of α- and β-thalassemia mutations in the Han people and other minority groups in Hainan Province. As shown in Table 6, α-thalassemia genotypes were found in twelve ethnic minorities (the Han, Li, Miao, Hui, Zhuang, Yao, Dong, Hani, Dai, Buyi, Tu, and Chuanqingren people). The α-thalassemia genotypes were most commonly found in the Li (73.5%), the Han (24.5%), and the Miao (1.1%) people, who account for 99.1% of α-thalassemia carriers. -α^3.7^/αα, -α^4.2^/αα, and -α^3.7^/-α^4.2^ were the most frequent α-thalassemia genotypes in the Li people, accounting for 17.4%, 16.9%, and 15.6% in the Li people, respectively; -α^4.2^/αα, -α^3.7^/αα, and --^SEA^/αα were the most frequent α-thalassemia genotypes in the Han people, accounting for 21.9, 20.1, and 18.2% in the Han people, respectively; αα^(WS)^/αα, -α^3.7^/αα and --^SEA^/αα were the most frequent α-thalassemia genotypes in the Miao people, accounting for 24.2, 21.2, and 21.2% in the Miao people, respectively.

**Table 6:** Distribution of α-thalassemia genotypes in the Han people and ethnic minorities in the southern area of Hainan.

**Genotype**	**Li**		**Han**		**Miao**		**Hui**		**Zhuang**		**Yao**		**Dai**		**Tujia**		**Buyi**		**Dong**		**Hani**		**Chuanqing**	
	**Count**	**Ratio**	**Count**	**Ratio**	**Count**	**Ratio**	**Count**	**Ratio**	**Count**	**Ratio**	**Count**	**Ratio**	**Count**	**Ratio**	**Count**	**Ratio**	**Count**	**Ratio**	**Count**	**Ratio**	**Count**	**Ratio**	**Count**	**Ratio**
		**(%)**		**(%)**		**(%)**		**(%)**		**(%)**		**(%)**		**(%)**		**(%)**		**(%)**		**(%)**		**(%)**		**(%)**
-α^4.2^/αα	743	17.4	313	21.9	12	18.2	2	6.7	1	12.5	1	16.7	0	0	0	0	0	0	0	0	0	0	0	0
-α^3.7^/αα	721	16.9	287	20.1	14	21.2	13	43.3	0	0	2	33.3	1	100.0	1	100.0	0	0	0	0	0	0	0	0
-α^3.7^/-α^4.2^	665	15.6	89	6.2	0	0	0	0	0	0	0	0	0	0	0	0	0	0	0	0	0	0	0	0
α^WS^/αα	436	10.2	141	9.9	16	24.2	11	36.7	2	25.0	1	16.7	0	0	0	0	0	0	0	0	0	0	0	0
α^WS/^-α^3.7^	362	8.5	59	4.1	1	1.5	0	0	0	0	0	0	0	0	0	0	0	0	0	0	0	0	0	0
-α^3.7^/-α^3.7^	331	7.8	43	3.0	4	6.1	1	3.3	0	0	0	0	0	0	0	0	0	0	0	0	0	0	0	0
α^WS^/-α^4.2^	311	7.3	41	2.9	0	0	1	3.3	0	0	0	0	0	0	0	0	0	0	0	0	0	0	0	0
-α^4.2^/-α^4.2^	281	6.6	43	3.0	0	0	0	0	0	0	0	0	0	0	0	0	0	0	0	0	0	0	0	0
α^WS^/α^WS^	103	2.4	13	0.9	0	0	0	0	0	0	0	0	0	0	0	0	0	0	0	0	0	0	1	0
α^QS/^αα	76	1.8	61	4.3	2	3.0	0	0	0	0	0	0	0	0	0	0	0	0	0	0	0	0	0	100.0
--^SEA^/αα	69	1.6	260	18.2	14	21.2	2	6.7	4	50.0	0	0	0	0	0	0	0	0	1	100.0	1	100.0	0	0
α^QS^/-α^3.7^	40	0.9	9	0.6	0	0	0	0	0	0	0	0	0	0	0	0	0	0	0	0	0	0	0	0
α^QS^/-α^4.2^	36	0.8	8	0.6	0	0	0	0	0	0	0	0	0	0	0	0	0	0	0	0	0	0	0	0
-α^3.7^/--^SEA^	33	0.8	18	1.3	1	1.5	0	0	0	0	0	0	0	0	0	0	0	0	0	0	0	0	0	0
-α^4.2^/--^SEA^	30	0.7	19	1.3	1	1.5	0	0	0	0	0	0	0	0	0	0	0	0	0	0	0	0	0	0
α^QS^/α^WS^	17	0.4	4	0.3	0	0	0	0	0	0	0	0	0	0	0	0	0	0	0	0	0	0	0	0
α^WS^/--^SEA^	11	0.3	8	0.6	1	1.5	0	0	0	0	0	0	0	0	0	0	0	0	0	0	0	0	0	0
α^CS^/αα	3	0.1	6	0.4	0	0	0	0	1	12.5	2	33.3	0	0	0	0	1	100.0	0	0	0	0	0	0
α^CS/^-α^4.2^	1	0.02	1	0.1	0	0	0	0	0	0	0	0	0	0	0	0	0	0	0	0	0	0	0	0
α^QS^/--^SEA^	1	0.02	0	0.0	0	0	0	0	0	0	0	0	0	0	0	0	0	0	0	0	0	0	0	0
α^CS^/--^SEA^	0	0	3	0.2	0	0	0	0	0	0	0	0	0	0	0	0	0	0	0	0	0	0	0	0
Total	4,270	73.5	1,426	24.5	66	1.1	30	0.5	8	0.1	6	0.1	1	0.02	1	0.02	1	0.02	1	0.02	1	0.02	1	0.02

β-Thalassemia genotypes were identified in six ethnic minorities (the Han, Li, Miao, Zhuang, Hui, and Yao people) (Table 7). The β-thalassemia genotypes were most commonly found in the Han (59.3%), the Li (31.7%), and the Miao (4.3%) patients, accounting for 95.4% of β-thalassemia carriers. β^CD41−42^/β^N^ was the dominant β-thalassemia genotype among the Han, Li, Miao, and Zhuang people, accounting for 61.2, 87.2, 81.3, and 60.0%, respectively (Table 7).

**Table 7:** Distribution of β-thalassemia genotypes in the Han people and ethnic minorities in the southern area of Hainan.

**Genotype**	**Li**		**Han**		**Miao**		**Hui**		**Zhuang**		**Yao**	
	**Count**	**Ratio (%)**	**Count**	**Ratio (%)**	**Count**	**Ratio (%)**	**Count**	**Ratio (%)**	**Count**	**Ratio (%)**	**Count**	**Ratio (%)**
β^41−42M^/β^N^	102	87.2	134	61.2	13	81.3	2	40.0	6	60.0	0	0
β^−28M^/β^N^	2	1.7	24	11.0	0	0	2	40.0	0	0	0	0
β^654M^/β^N^	3	2.6	19	8.7	0	0	0	0	0	0	1	50.0
β^71−72M^/β^N^	3	2.6	14	6.4	0	0	0	0	0	0	0	0
β^E^/β^N^	5	4.3	10	4.6	0	0	0	0	2	20.0	0	0
β^17M^/β^N^	1	0.9	9	4.1	2	12.5	0	0	2	20.0	0	0
β^IntM^/β^N^	0	0	3	1.4	0	0	0	0	0	0	0	0
β^IVS−I−1M^/β^N^	0	0	1	0.5	1	6.3	0	0	0	0	1	50.0
β^27/28M^/β^N^	0	0	1	0.5	0	0	1	20.0	0	0	0	0
β^−29M^/β^N^	0	0	2	0.9	0	0	0	0	0	0	0	0
β^43M^/β^N^	0	0	1	0.5	0	0	0	0	0	0	0	0
β^CAPM^/β^N^	0	0	1	0.5	0	0	0	0	0	0	0	0
β^41−42M^/β^41−42M^	1	0.9	0	0	0	0	0	0	0	0	0	0
Total	117	31.7	219	59.3	16	4.3	5	1.4	10	2.7	2	0.5

Among the subjects carrying both α and β-thalassemia variations, only three ethnic minorities were identified, including the Li, Han, and Miao people, accounting for 82.0, 17.4, and 0.7%, respectively (Table 8). -α^3.7^/αα, -α^4.2^/αα, and -α^3.7^/-α^4.2^ were the three most frequent genotypes in the Li people, all of which were accompanied by β^CD41−42^/β^N^, accounting for 18.6, 17.6, and 14.1%, respectively. -α^4.2^/αα, -α^3.7^/αα, and α^WS^/αα were the three most frequent genotypes in the Han people, all of which were accompanied by β^CD41−42^/β^N^, accounting for 23.3, 19.4, and 13.2%, respectively. The Miao people who carried -α^4.2^/αα, -α^3.7^/αα, α^WS^/αα, and --^SEA^/αα accompanied by β^CD41−42^/β^N^ accounted for <1% of the total subjects (Table 8).

**Table 8:** Distribution of α-composite β-thalassemia genotypes in the Han people and ethnic minorities in the southern area of Hainan.

**Genotype**	**Li**		**Han**		**Miao**	
	**Count**	**Ratio (%)**	**Count**	**Ratio (%)**	**Count**	**Ratio (%)**
-α^3.7^/αα & β^41−42M^/β^N^	113	18.6	25	19.4	1	20.0
-α^4.2^/αα & β^41−42M^/β^N^	107	17.6	30	23.3	2	40.0
-α^3.7^/-α^4.2^ & β^41−42M^/β^N^	86	14.1	13	10.1	0	0
α^WS^/αα & β^41−42M^/β^N^	74	12.2	17	13.2	1	20.0
α^WS^/-α^3.7^ & β^41−42M^/β^N^	48	7.9	3	2.3	0	0
α^WS^/-α^4.2^ & β^41−42M^/β^N^	44	7.2	7	5.4	0	0
-α^4.2^/-α^4.2^ & β^41−42M^/β^N^	43	7.1	4	3.1	0	0
-α^3.7^/-α^3.7^ & β^41−42M^/β^N^	40	6.6	4	3.1	0	0
α^QS^/-α^3.7^ & β^41−42M^/β^N^	10	1.6	0	0	0	0
α^WS^/α^WS^ & β^41−42M^/β^N^	7	1.1	1	0.8	0	0
α^QS^ /-α^4.2^ & β^41−42M^/β^N^	5	0.8	0	0	0	0
--^SEA^/αα & β^41−42M^/βN	4	0.7	7	5.4	1	20.0
α^QS^/α^WS^ & β^41−42M^/β^N^	4	0.7	1	0.8	0	0
α^QS^ /αα & β^41−42M^/βN	4	0.7	0	0	0	0
α^WS^/--^SEA^ & β^41−42M^/β^N^	3	0.5	0	0	0	0
-α^3.7^/--^SEA^ & β^41−42M^/β^N^	2	0.3	1	0.8	0	0
α^WS^/αα & β^−28M^/β^N^	2	0.3	1	0.8	0	0
-α^4.2^/--^SEA^ & β^41−42M^/β^N^	2	0.3	0	0	0	0
-α^4.2^/αα & β^654M^/β^N^	2	0.3	0	0	0	0
-α^3.7^/αα & β^41−42M^/β^41−42M^	2	0.3	0	0	0	0
-α^3.7^/αα & β^71−72M^/β^N^	1	0.2	1	0.8	0	0
α^CS^/αα & β^41−42M^/β^N^	1	0.2	1	0.8	0	0
-α^3.7^/αα & β^−28M^/β^N^	1	0.2	0	0	0	0
-α^4.2^/-α^4.2^ & β^71−72M^/β^N^	1	0.2	0	0	0	0
α^WS^/αα & β^654M^/β^N^	1	0.2	0	0	0	0
α^WS^/α^QS^ & β^41−42M^/β^N^	1	0.2	0	0	0	0
α^WS^/-α^4.2^ & β^41−42M^/β^41−42*M*^	1	0.2	0	0	0	0
-α^4.2^/αα & β^71−72M^/βN	0	0	3	2.3	0	0
--^SEA^/αα & β^17M^/β^N^	0	0	2	1.6	0	0
--^SEA^/αα & β^71−72M^/β^N^	0	0	2	1.6	0	0
-α^3.7^/αα & β^17M^/β^N^	0	0	1	0.8	0	0
-α^3.7^/αα & β^43M^/β^N^	0	0	1	0.8	0	0
-α^3.7^/αα & β^654M^/β^N^	0	0	1	0.8	0	0
-α^4.2^/αα & β^IntM^/β^N^	0	0	1	0.8	0	0
α^WS^/-α^3.7^ & β^71−72M^/β^N^	0	0	1	0.8	0	0
α^WS^/αα & β^71−72M^/β^N^	0	0	1	0.8	0	0
Total	609	82.0	129	17.4	5	0.7

## Discussion

Hainan Province has a high incidence of thalassemia, second only to Guangxi and Guangdong Province in China. In this study, we reported the molecular characteristics, ethnic distribution, and geographical distribution of thalassemias in the southern area of Hainan Province. A total of 6,924 thalassemia-positive subjects (70.6%) were identified among 9,813 suspected carriers of thalassemia in our hospital. Of the 6,924 thalassemia-positive subjects, 83.9% were α-thalassemia mutation carriers, 5.3% were β-thalassemia mutation carriers, and 10.7% were both α- and β-thalassemia mutation carriers. The results revealed that the carrier frequency was higher than those in previous reports in other provinces of China, such as Guangdong (11.6%) (12), Sichuan (3.4%) (13), Dongguan (Guangdong) (47.4%) (14), Chongqing (7.8%) (15), Wuhan (57.3%) (16), Jiangxi (50.3%) (17), Guangxi (50.1%) (18), Guangxi (67.0%) (19), Yunnan (31.9%) (20), and Hubei (34.9%) (21), but lower than those described in Yunan (84.2%) (22), Baise (Guangxi) (92.5%) (23), and Yulin (Guangxi) (87.1%) (24). This difference was probably due to the screening strategy. In the present study, the subjects were mainly selected as suspected thalassemia carriers, and most of them had microcytosis, while in some studies (12, 13), healthy subjects undergoing routine genetic screening were also included.

The genotype distribution in the current study was different from those in previous reports, as the proportion of the α-thalassemia mutation was nearly sixteen times that of the β-thalassemia mutation. The frequencies of α- and β-thalassemia were 83.9% and 5.3% in the total subjects in our study, respectively. Previous studies have shown that α-thalassemia is mainly caused by three types of gene deletions, including -α^3.7^, -α^4.2^, and --^SEA^. In the current study, eight α-thalassemia deletion genotypes were identified, including -α^3.7^/αα, -α^4.2^/αα, -α^3.7^/-α^4.2^, -α^3.7^/-α^3.7^, --^SEA^/αα, -α^4.2^/-α^4.2^, -α^3.7^/--^SEA^, and -α^4.2^/--^SEA^, which accounted for 69.2% of all α-thalassemia mutations in carriers. -α^3.7^ and -α^4.2^ deletions were the most common deletions in the southern part of Hainan. However, the --^SEA^ deletion was the most common α-thalassemia genotype in the southern region of China, such as Guangdong (14, 25), Guangxi (18, 19, 23, 24, 26), Yunnan (20, 22), Sichuan (13), Jiangxi (17), Hubei (16, 21) and Hunan Province (27). Wang et al. (11) reported that --^SEA^/αα was identified in 39.1% of α-thalassemia mutation carriers in Hainan. However, --^SEA^/αα was identified only in 6.0% of all α-thalassemia mutation carriers in the southern area of Hainan. Ethnic differences may also play a significant role in genetic differences, so we also analyzed thalassemia genotypes in the Han and the minority ethnic groups. In the present study, 74.1% of --^SEA^/αα carriers were the Han people, and 19.7% were the Li people, which is consistent with previous studies (11, 28), indicating that the --^SEA^/αα mutation is more common in the Han than the Li people.

β-Thalassemias are mainly caused by point mutations, while a few of them are caused by gene deletions. At present, 945 mutation types of β-thalassemia have been identified worldwide, and over 145 different β- thalassemia gene mutations have been detected in China. The most common mutations are β^CD41−42^, β^CD17^, β^IVS−II−654^, β^−28*M*^, β^CD71−72^, β^−26*M*^, β^IVS−I−1^, and β^−29*M*^, and these mutations account for more than 95.0% of all β mutations found in the Chinese population (29). In the current study, 13 genotypes were detected, and β^CD41−42^/β^N^ (69.6%) was the most common β-thalassemia genotype in the southern part of Hainan, which was similar to that in an earlier study (28) and different from that in another study (11). The proportion of β^CD41−42^ (88.0%) among β-thalassemia in the southern part of Hainan was the highest and was higher than those in other provinces of China (12, 16, 17, 30). In the Li people, nearly 97.0% of β-thalassemia mutations were β^CD41−42^, whereas in the Han population, 71.3% of β-thalassemia mutations were β^CD41−42^. The β^IVS−2−654^ mutation was identified in 2.4% of β-thalassemia patients in the southern part of Hainan, which was similar to that in Guangxi Province (2.7%) (23). However, it was the most common mutation in Wuhan (41.2%) (16), Fujian (41.2%) (30), and Hunan (33.7%) (27). β^CD17^ was detected in 1.5% of β-thalassemia cases in the southern part of Hainan, which was lower than that in other provinces of southern China, such as Guangdong (12), Guangxi (18, 19, 23), Yunnan (20, 22), and Sichuan (13). It is the most common mutation in Yunan (20), Guizhou (31), and Baise (Guangxi Province) (23). This result may be explained by the distinctive customs and identities of different ethnicities. Due to special geographical locations, customs, and cultures, the proportion of intermarriages between some regions of Hainan Province and other regions is relatively small, which consequently leads to an overlapping distribution of thalassemia genes and results in a high number of thalassemia carriers. Compared with other countries, the most frequent genotype of β-thalassemia was similar to that in Thailand (23), while it was different from Pakistan (24), Syria (25), Iran (20), Vietnam (26) Malaysia (27), and India (28).

Among α-thalassemia mutations, -α^4.2^ was the most common mutation in Ledong, Lingshui, Baoting, and Wuzhishan, -α^3.7^ was the most common mutation in Sanya, which was different from those in previous research in the corresponding city of Hainan Province (11). Among the β-thalassemia mutations, β^CD41−42^ accounted for the largest proportion among the five regions, which is consistent with a previous study that reported it to be the most frequent genotype in Sanya, Ledong, Lingshui, Baoting, and Wuzhishan of Hainan Province (11). These findings may be explained by the migration of the population and the special location of Hainan Province, which is located at the southeastern end of China.

## Conclusion

The genotypes of α- and β-thalassemia in Hainan Province in China are characterized by a wide distribution, a high carrier rate, genetic heterogeneity, significant geographical differences and ethnic specificity. This study enriches the knowledge of the thalassemia mutation spectrum in the Chinese people and provides valuable information for genetic counseling, prenatal diagnosis, and prevention of thalassemia in Hainan Province. We also recommend further studies on contributing factors that can affect the accessibility of thalassemia interventions to provide a scientific basis for government decision-making.

## Data Availability Statement

The raw data supporting the conclusions of this article will be made available by the authors, without undue reservation.

## Ethics Statement

The studies involving human participants were reviewed and approved by the Ethics Committee of Sanya Women and Children's Hospital. The patients/participants provided their written informed consent to participate in this study.

## Author Contributions

GY, ZL, and HW conceived and designed the project. YY, CJL, YG, CYL, DL, JW, and HW collected the samples and clinical information. YY and GY analyzed and interpreted the data. GY wrote the manuscript. All authors read and approved the final manuscript.

## Funding

The project was supported by grant from the Health Commission of Hainan Province, China (20A200237), grant from Hainan Province Clinical Medical Center, and the Golden Coconut Seed Funding (JYZZ-ZD-202102).

## Conflict of Interest

The authors declare that the research was conducted in the absence of any commercial or financial relationships that could be construed as a potential conflict of interest.

## Publisher's Note

All claims expressed in this article are solely those of the authors and do not necessarily represent those of their affiliated organizations, or those of the publisher, the editors and the reviewers. Any product that may be evaluated in this article, or claim that may be made by its manufacturer, is not guaranteed or endorsed by the publisher.
