# Supplementary material for: Molecular Spectrum, Ethnic and Geographical Distribution of Thalassemia in the Southern Area of Hainan, China
Source: Front Pediatr. 2022 Jun 10;10:894444. doi: 10.3389/fped.2022.894444 (PMC9245522; doi:10.3389/fped.2022.894444)
Supplement: Supplementary file 1 [file Table_1.DOCX]

Supplementary Table 1. Hematological data of patients with α-thalassemia genotypes (n ≥ 5)

| Genotype | Count (n) | Hb (g/L) | MCV (fL) | MCH (pg) |
| --- | --- | --- | --- | --- |
| -α^4.2^/αα | 1072 | 130.0±17.9 | 79.0±4.2 | 26.0±2.1 |
| -α^3.7^/αα | 1039 | 129.4±18.0 | 79.2±4.4 | 26.1±2.4 |
| -α^3.7^/-α^4.2^ | 754 | 128.0±18.0 | 72.9±4.1 | 23.3±1.6 |
| α^WS^/αα | 607 | 127.0±21.0 | 79.2±4.8 | 26.2±2.5 |
| α^WS/^-α^3.7^ | 422 | 131.4±19.6 | 78.0±4.3 | 25.4±2.3 |
| -α^3.7^/-α^3.7^ | 379 | 127.8±18.8 | 73.0±4.2 | 23.5±1.9 |
| α^WS^/-α^4.2^ | 353 | 129.5±18.9 | 78.0±5.0 | 25.3±2.3 |
| --^SEA^/αα | 351 | 123.6±17.4 | 69.1±4.0 | 21.8±2.1 |
| -α^4.2^/-α^4.2^ | 324 | 127.6±18.1 | 72.7±4.0 | 23.1±1.5 |
| α^QS/^αα | 140 | 130.3±18.3 | 76.3±3.9 | 24.6±2.0 |
| α^WS^/α^WS^ | 116 | 134.1±19.3 | 80.3±3.4 | 26.1±1.4 |
| -α^3.7^/--^SEA^ | 52 | 95.9±16.9 | 60.7±6.3 | 18.5±2.4 |
| -α^4.2^/--^SEA^ | 50 | 96.5±16.4 | 61.3±6.9 | 18.5±1.9 |
| α^QS^/-α^3.7^ | 49 | 117.6±16.0 | 66.1±3.9 | 21.6±2.3 |
| α^QS^/-α^4.2^ | 44 | 121.4±14.2 | 66.1±4.2 | 20.8±1.5 |
| α^QS^/α^WS^ | 21 | 131.4±20.0 | 73.9±4.5 | 24.1±2.6 |
| α^WS^/--^SEA^ | 20 | 119.9±17.3 | 66.5±4.0 | 20.0±1.8 |
| α^CS^/αα | 13 | 124.1±18.6 | 76.1±6.1 | 25.5±3.9 |

Supplementary Table 2. Hematological data of patients with β-thalassemia genotypes (n ≥ 5)

| Genotype | Count (n) | Hb (g/L) | MCV (fL) | MCH (pg) |
| --- | --- | --- | --- | --- |
| β^41–42M^/β^N^ | 257 | 116.8±16.1 | 64.3±3.8 | 20.4±1.8 |
| β^–28M^/β^N^ | 28 | 124.7±14.8 | 72.6±4.1 | 23.3±1.6 |
| β^654M^/β^N^ | 23 | 119.4±15.1 | 65.5±3.5 | 21.3±2.5 |
| β^71–72M^/β^N^ | 17 | 112.5±18.1 | 64.4±2.8 | 20.2±0.8 |
| β^E^/β^N^ | 17 | 125.6±15.8 | 75.9±3.7 | 24.3±2.8 |
| β^17M^/β^N^ | 14 | 121.3±20.6 | 65.9±3.8 | 20.7±2.1 |

Supplementary Table 3. Hematological data of patients with α-composite β-thalassemia genotypes (n ≥ 5)

| Genotype | Count (n) | Hb (g/L) | MCV (fL) | MCH (pg) |
| --- | --- | --- | --- | --- |
| -α^3.7^/αα & β^41-42M^/β^N^ | 139 | 123.5±17.1 | 67.5±3.8 | 21.8±1.4 |
| -α^4.2^/αα & β^41–42M^/β^N^ | 139 | 121.7±19.0 | 68.0±4.4 | 21.7±1.9 |
| -α^3.7^/-α^4.2^ & β^41–42M^/β^N^ | 99 | 126.0±19.2 | 74.6±4.6 | 24.1±1.9 |
| α^WS^/αα & β^41–42M^/β^N^ | 92 | 116.4±17.8 | 64.8±4.0 | 20.6±2.0 |
| α^WS^/-α^3.7^ & β^41–42M^/β^N^ | 51 | 128.7±14.7 | 70.2±4.3 | 22.3±1.4 |
| α^WS^/-α^4.2^ & β^41–42M^/β^N^ | 51 | 124.1±18.9 | 69.0±4.9 | 22.3±1.8 |
| -α^4.2^/-α^4.2^ & β^41–42M^/β^N^ | 47 | 129.0±18.1 | 75.4±3.9 | 24.5±1.7 |
| -α^3.7^/-α^3.7^ & β^41–42M^/β^N^ | 44 | 124.5±14.7 | 74.9±4.2 | 23.9±2.0 |
| --^SEA^/αα & β^41–42M^/βN | 12 | 123.3±33.9 | 72.6±3.4 | 23.5±0.8 |
| α^QS^/-α^3.7^ & β^41–42M^/β^N^ | 10 | 121.3±17.0 | 66.1±3.6 | 20.8±1.1 |
| α^WS^/α^WS^ & β^41–42M^/β^N^ | 8 | 124.3±16.9 | 64.2±9.7 | 21.3±1.5 |
| α^QS^ /-α^4.2^ & β^41–42M^/β^N^ | 5 | 124.2±18.1 | 66.1±1.4 | 21.4±0.7 |
| α^QS^/α^WS^ & β^41–42M^/β^N^ | 5 | 123.2±11.6 | 65.3±3.5 | 21.4±0.9 |
